# Predictors of saccadic reaction time among young children in Lusaka, Zambia

**DOI:** 10.1371/journal.pone.0339819

**Published:** 2026-03-26

**Authors:** Jacqueline M. Lauer, Juha Pyykkö, Anthony Consigli, Mpela Chembe, Tamara Billima-Mulenga, Savanna Henderson, Doug Parkerson, Jukka M. Leppänen, Lindsey M. Locks, Günther Fink, Peter C. Rockers

**Affiliations:** 1 Department of Health Sciences, Sargent College of Health & Rehabilitation Sciences, Boston University, Boston, Massachusetts, United States of America; 2 Department of Global Health, Boston University School of Public Health, Boston, Massachusetts, United States of America; 3 Department of Epidemiology, Boston University School of Public Health, Boston, Massachusetts, United States of America; 4 Innovations for Poverty Action Zambia, Lusaka, Zambia; 5 Innovations for Poverty Action, Washington, DC, United States of America; 6 Department of Psychology and Speech-Language Pathology, University of Turku, Turku, Finland; 7 University of Basel and Swiss Tropical and Public Health Institute, Basel, Switzerland; PLOS ONE, UNITED KINGDOM OF GREAT BRITAIN AND NORTHERN IRELAND

## Abstract

Saccadic reaction time (SRT), an assessment of visual processing speed, may afford an accurate and unbiased measure of early childhood development (ECD). Few studies have examined SRT in low- and middle-income countries (LMICs), including its drivers. We sought to identify predictors of SRT as well as to assess the correlation between SRT and concurrent measures of ECD [Global Scales of Early Development (GSED) development-for-age Z-score (DAZ), height-for-age Z-score (HAZ), and head circumference-for-age Z-score (HCZ)], among young children in Lusaka, Zambia. We conducted a sub-study among 299 Lusakan children participating in a 2x2 cluster-randomized trial. SRT was assessed at ~31 months using a screen-based setup with a Tobii Pro Fusion tracker. Associations with household, caregiver, and child characteristics were assessed using univariable regression models; predictors significant at the p < 0.20 level were retained in a multivariable model. Pearson correlation coefficients were calculated to assess associations between SRT and other concurrent measures of ECD. In the multivariable model, characteristics found to be significant predictors of SRT included: being the only child <5 in the household at baseline (β: −10.02, 95% CI: −19.71, −0.33, p = 0.04), length-for-age Z-score (LAZ) at baseline (β: −3.17, 95% CI: −6.31, −0.04, p = 0.047), consuming ≥4 food groups in the past day (β: −10.42, 95% CI: −19.98, −0.86, p = 0.03), and having diarrhea in the past 2 weeks (β: 12.38, 95% CI: 0.71, 24.06, p = 0.04). SRT was significantly negatively correlated with HAZ (−0.176, p < 0.01) and HCZ (−0.132, p < 0.05), but not GSED DAZ. Overall, we identified several significant predictors of SRT among young children in Lusaka, Zambia, including birth spacing, baseline LAZ, dietary diversity, and diarrheal disease. Further research is needed, including in different age groups and geographic locations, to better understand the drivers of slow SRT, and poor ECD generally, in LMICs.

## Introduction

Early childhood development (ECD), encompassing the physical, cognitive, motor, language, social, and emotional development of children in the early years of life, is associated with a myriad of positive health, economic, and social outcomes later in life [1]. It was previously estimated, using proxy measures of stunting and poverty, that 43% – ~ 250 million – of children <5 years of age in low- and middle-income countries (LMICs) were at risk of not reaching their developmental potential [2]. In 2015, ECD became part of the United Nations (UN) Sustainable Development Goals (SDGs) [3], underscoring the need for measurements of ECD that are feasible, reliable, valid, and comparable across countries. However, to date, quantification is challenging as there remains no established global standards for measuring poor ECD [4].

Given difficulties assessing ECD, linear growth (or stunting) remains a common proxy measure for cognitive development among infants and young children [2,5,6]. However, while linear growth and cognitive development share a number of common determinants, (e.g., inadequate nutrition, exposure to infectious diseases, and insufficient social and cognitive stimulation) [6,7], associations between the two have proven inconsistent in the literature [8,9]. Furthermore, results from a meta-analysis of randomized trials demonstrate that intervention effects on child growth and ECD do not always co-occur, further suggesting that using child growth as a proxy for ECD may be inadequate [10]. At the same time, while observational assessments, such as Bayley Scales of Infant and Toddler Development and the Global Scales for Early Development (GSED) Long Form, offer a more direct measure of ECD, they can be time consuming, resource-intensive, and potentially culturally biased [11].

Compared with linear growth and observational assessments, eye-tracking may offer a more accurate, less biased, and more widely applicable measure of child cognitive development [12]. Within eye-tracking, saccadic reaction time (SRT), a measure of the speed of visual and oculomotor processing, refers to the time it takes for the eyes to initiate an eye movement (saccade) after a visual stimulus appears, typically measured in milliseconds (ms). Faster visuomotor speed is indicative of advanced underlying neural maturation and white matter integrity [13,14], and studies from high-income countries have linked faster SRT to future developmental outcomes, including stronger cognitive and executive function skills later in life [15,16].

Despite the fact that the majority of eye-tracking studies have been conducted in high-income countries, several recent studies have established feasibility in LMICs as well. In a study of 9-month-old Malawian and Finnish infants, completion rates for the whole-eye tracking test were similar in the two populations (Finland 95%, Malawi 90%), and a high percentage of parents in Malawi (92%) found the method of assessment to be acceptable [17]. In The Gambia, various eye-tracking tasks were assessed with under 2-year-old infants, and the results provided similar data quality as infants from the United Kingdom [18].

It has been observed that mean SRTs are slower in children from LMICs vs. high-income countries [17]. Furthermore, a study in South Africa and Zambia found SRTs were slower in children from households with lower wealth indices [19]. However, there is an overall dearth of studies examining the predictors of SRT in young children, particularly in LMICs. In this secondary analysis of a randomized controlled trial, we sought to examine the predictors of SRT as well as assess the correlation between SRT and other concurrent measures of ECD, including the GSED development-for-age Z-score (DAZ), height-for-age Z-score (HAZ), and head circumference-for-age Z-score (HCZ), among young children in Lusaka, Zambia.

## Materials and methods

### Description of parent trial

Participants in this sub-study were drawn from a 2x2 cluster-randomized factorial trial (ClinicalTrials.gov identifier: NCT05120427) conducted in 3 districts in Zambia between 2021 and 2023, with the overall goal of assessing the effect of growth charts as well as small-quantity lipid-based nutrient supplements (SQ-LNS) on growth [20]. In the parent trial, 2,291 caregiver-child pairs from 282 communities were randomly assigned in equal proportions (1:1:1:1) to 1 of 4 groups: (1) home-installed growth charts, (2) monthly provision of SQ-LNS, (3) a combination of growth charts and SQ-LNS, or (4) no intervention. All caregiver-child pairs were eligible for inclusion if the child was between 2 and 10 months old at baseline, at least 6 months old when the intervention began, and if written consent was provided. Participants were excluded if they indicated plans to relocate outside the study area within 12 months of recruitment.

At both baseline and endline, enumerators were trained to identify the primary caregiver of the index child to conduct a questionnaire. Caregivers were interviewed about household composition, sociodemographic factors, infant and young child feeding (IYCF) practices, child morbidity, and engagement with the index child. After the questionnaire was completed, the child’s length/height and weight were assessed. At baseline, length was measured to the nearest 0.1 cm in a recumbent position using a length board. At endline, height was measured to the nearest 0.1 cm in a standing position using a stadiometer (Seca 217, Seca, Hamburg, Germany). Weight was recorded with a 5-gram precision using a digital scale (Seca 874, Seca, Hamburg, Germany). Height and weight were each measured at least twice by the same assessor, with additional measurements taken if the difference exceeded 0.7 cm for length/height or 100 g for weight. At endline, study staff also assessed head circumference and the GSED Long Form [21].

### Description of biomarkers sub-study

After completing the endline questionnaire, between 27-06-2023 and 13-09-2023, study participants from Lusaka district were invited to take part in a biomarkers sub-study, which included an eye-tracking assessment at Chawama First Level Hospital. A total of 318 children participated in the biomarkers sub-study, with 299 of them successfully completing the eye-tracking assessment. This sample size provides 99% power to detect a standardized effect size of 0.15 (medium), assuming α = 0.05 and 13 predictors (G*Power software, Düsseldorf, Germany) [22].

### Description of eye-tracking assessment

The eye-tracking assessment was performed in a room where the eye-tracking section was separated with black curtains to ensure privacy and reduce visual distractions. For the purposes of the assessment, the child was seated on their caregiver’s lap with their eyes facing forward, positioned at a viewing distance of approximately 60–70 cm from the screen-based eye tracker (Tobii Pro Fusion, Tobii AB, Stockholm, Sweden) and the 24-inch monitor (60 Hz, aspect ratio 16:9, resolution 1920 x 1080 pixels, display area 531.36 x 298.89 mm). The child was then shown short, alternating blocks of visual stimuli on the monitor, designed to calibrate the eye-tracking system, record fixations on social scenes, and measure SRT. In the SRT task, saccade targets consisted of sinusoidal grating stimuli with a Gaussian mask (size 5.0° x 5.0°). The saccade target was shown in the position for 1.5 sec before reappearing at a randomly selected on-screen location, with an oval distance (324–576 pixels, 8.55–15.13°, on a circle with 16:9 aspect ratio) between 2 subsequent targets.

To capture the children’s attention with the targets, the test began with a colorful GIF of a cartoon fish accompanied by a bubble sound for 2.0 secs in the center of the screen, before transitioning into a saccade target in the same position. After an initial stimulus in the center of the screen (which was not analyzed), the child viewed a total of 11 saccade targets in each session, with 6 sessions in total to accumulate enough saccades to reliably estimate the mean SRT. The fish GIF was shown 3 times in the same position as the randomly selected saccade targets during each session, with different fish cartoons in each session. Videos of social scenes were preceded by each SRT session.

Gaze data were recorded at 120 Hz and filtered with a 15-sample (125 ms) median filter to eliminate abrupt spikes. Gaze locations were calculated by averaging the xy-coordinates from both eyes, or, if data for 1 eye were missing, by using the xy-coordinates from the valid eye. Gaze was considered valid if at least 1 eye was recorded. A valid SRT was defined as the time interval from the onset of the saccade target to the last time point in the previous saccade target for saccades which reached the new saccade target, were not affected by excessive missing data or an excessively long gaze travel time (i.e., > 100 ms of continuous missing data during the period starting at the onset of the saccade target and ending at the registration of the gaze in the new target, a missing sample preceding the gaze entry to the new target, or >75 ms travel time between targets), and fell inside a visually inspected time window starting 150 ms after target onset and ending 650 ms after target onset (outliers excluded 2.1% of otherwise valid SRTs between 100–1,000 ms). A small margin (0.9°, 34 pixels) was added to the area of saccade targets to account for calibration errors. Participants were required to have ≥5 valid SRTs to be included in the analyses.

### Data preparation and statistical analyses

Anthropometric measurements were converted to Z‐scores using the World Health Organization (WHO) standards, and outliers, based on biologically implausible values, were excluded [23,24]. Stunted, wasted, and underweight were defined as <−2 SDs for length/height-for-age Z-score (LAZ/HAZ), weight-for-length/height Z-score (WLZ/WHZ), and weight-for-age Z-score (WAZ), respectively. Responses to the GSED were used to calculate a child’s raw developmental score (D-score) and a normalized development-for-age Z-score (DAZ) [25]. Child dietary diversity was assessed by summing 7 food groups: grains, roots, and tubers; pulses, nuts, and seeds; dairy products; flesh foods; eggs; vitamin-A-rich fruits and vegetables; and other fruits and vegetables. A binary indicator was created based on the consumption of 4 or more food groups in the past day [26]. Finally, a proxy indicator for caregiver engagement was created based on engagement in the following 6 activities in the past 3 days: reading books, telling stories, singing songs, going outside the home, playing, and naming/counting/drawing.

Descriptive statistics, including means with standard deviations and frequencies with percentages, were calculated to describe participant characteristics. All household, caregiver, and child predictors had > 95% complete data; variables with missing values were excluded from the analyses and are not listed in the tables, with the exception of “unknown” for child HIV exposure in utero. The decision to retain child sex, child age, and study group in the multivariable regression model was made a priori. All other predictors significant at the p < 0.20 level in univariable regression analyses were also retained in the multivariable regression model, which included 294 participants. For the multivariable regression model, a 2-tailed p-value <0.05 was considered statistically significant. Multicollinearity was examined in this model by calculating variance inflation factors (VIFs). Finally, Pearson correlation coefficients were calculated to assess the association between SRT and other measures of ECD at endline, including HAZ, head circumference-for-age Z-score (HCZ), and GSED DAZ. Statistical analyses were conducted using STATA 18 software (Stata Corp, College Station, TX).

### Approvals

Caregivers provided written informed consent before enrolling in the parent trial and the biomarkers sub-study. The study was approved by the University of Zambia Biomedical Research Ethics Committee and the Ethics Committee for Northwestern Switzerland (EKNZ).

## Results

**Fig 1:**
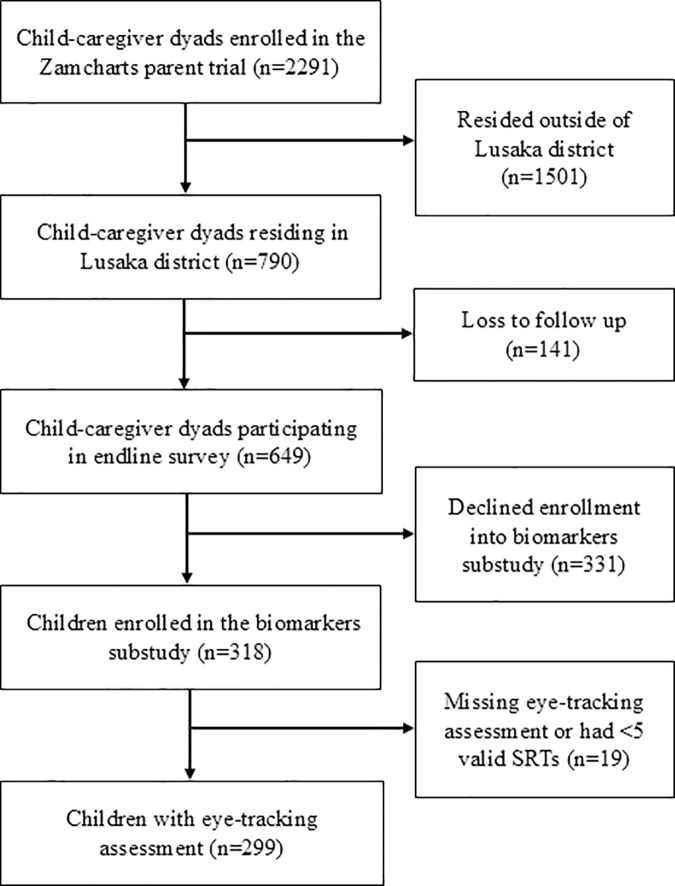
Flow diagram of study. SRT, saccadic reaction time**.**

The study flow diagram is presented in Fig 1. A total of 2,291 caregiver-child pairs were enrolled in the parent trial, and, of these, 790 resided in Lusaka district. A total of 649 participated in the endline questionnaire, and, of these, 318 participated in the biomarkers sub-study. The majority (n = 314) completed the eye-tracking assessment, and 299 had ≥ 5 valid SRTs. We found no statistically significant differences in household, caregiver, or child characteristics between children from Lusaka who participated in the biomarkers sub-study and those who did not.

### Descriptive statistics

**Fig 2:**
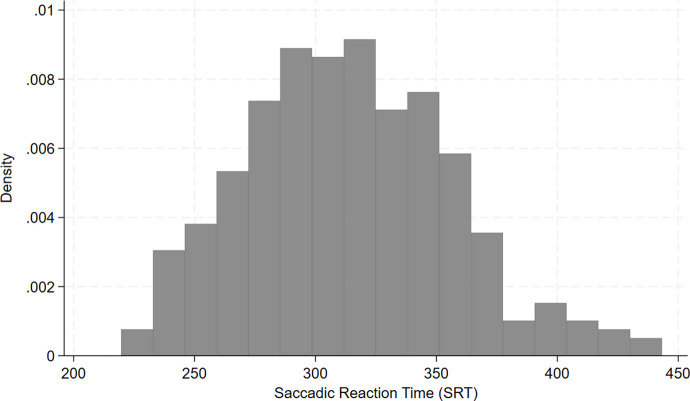
Distribution of saccadic reaction times for 299 children in Lusaka, Zambia.

Table 1 presents the descriptive characteristics for 299 Lusakan children. On average, households had 7 members, and, in half of households, the index child was the only child <5 years of age. Overwhelmingly, households had piped water (92%) and used charcoal or wood as a fuel source (84%). Caregivers were 32 years old, 71% were married, and 74% attended some secondary school or more. Just over half of children were female, 15% were born low birth weight (<2,500 g), and 7% were a twin. A quarter (26%) reported exclusive breastfeeding at 6 months of age, and 87% reported breastfeeding until ≥12 months of age. At endline, children were 31 months old and 61% consumed ≥4 food groups the previous day. In the previous 2 weeks, 27% of children had diarrhea, 47% had fever, and 67% had difficulty breathing. During the eye-tracking assessment, children had, on average, 21.1 *±* 9.2 valid SRTs. Fig 2 shows the distribution of SRTs in the sample.

**Table 1:** Characteristics of study participants and their association with saccadic reaction time among young children in Lusaka, Zambia.

	*Mean ± SD or n (%)* n = 299	*B (95% CI)*	*p-value*
**Household baseline characteristics**			
Members	6.5 ± 2.7	0.87 (−0.93, 2.67)	0.34
Child is only household member <5 years	151 (50.5)	−9.04 (−18.62, 0.55)	0.06*
Times with no food to eat, past 30 days			
0	162 (54.2)	ref	
1-2	54 (18.1)	−0.15 (−13.25, 12.96)	0.99
≥ 3	83 (27.8)	3.06 (−8.20, 14.32)	0.59
Water source			
Other	25 (8.4)	ref	
Piped	274 (91.6)	−12.77 (−30.12, 4.57)	0.15*
Fuel source			
Electricity	49 (16.4)	ref	
Charcoal/wood	250 (83.6)	14.47 (1.55, 27.38)	0.03*
Study group			
Control	66 (22.1)	ref	
Charts	80 (26.8)	9.01 (−4.80, 22.82)	0.20
SQ-LNS	71 (23.8)	11.60 (−2.60, 25.80)	0.11*
Charts + SQLNS	82 (27.4)	1.23 (−12.51, 14.95)	0.86
**Caregiver characteristics**			
Age, years	31.6 ± 9.1	0.24 (−0.29, 0.77)	0.37
Educational attainment			
Primary school or less	77 (26.2)	ref	
Junior secondary school	85 (28.9)	−6.83 (−19.87, 6.20)	0.30
Senior secondary school or more	132 (44.9)	−13.72 (−25.60, −1.83)	0.02*
Marital status			
Married	212 (70.9)	ref	
Divorced, separated, or widowed	49 (16.4)	9.65 (−3.48, 22.77)	0.15*
Never married	38 (12.7)	−10.12 (−24.71, 4.46)	0.17*
**Child characteristics**			
***Baseline characteristics***			
Age, months	5.0 ± 1.9	0.85 (−1.71, 3.42)	0.51
Sex, female	160 (53.5)	2.32 (−7.34, 11.98)	0.64
Twin	22 (7.4)	0.79 (−17.67, 19.25)	0.93
Birthweight, kg (n = 297)	2.94 ± 0.5	−3.41 (−12.34, 5.52)	0.45
Low birthweight, < 2,500 g	45 (15.2)	6.86 (−6.59, 20.32)	0.32
Exposed to HIV in utero			
No	223 (74.6)	ref	
Yes	57 (19.1)	5.50 (−6.87, 17.86)	0.38
Unknown	19 (6.4)	−4.57 (−24.48, 15.34)	0.65
Length-for-age Z-score (LAZ) (n = 294)	−0.70 ± 1.44	−3.64 (−6.99, −0.30)	0.03*
Stunted (LAZ < −2)	48 (16.3)	4.23 (−8.93, 17.38)	0.53
Weight-for-length Z-score (WLZ) (n = 294)	0.26 ± 1.37	−0.60 (−4.16, 2.95)	0.74
Wasted (WLZ < −2)	14 (4.8)	5.00 (−17.80, 27.80)	0.67
Weight-for-age Z-score (WAZ) (n = 296)	−0.38 ± 1.19	−3.65 (−7.71, 0.41)	0.08*
Underweight (WAZ < −2)	24 (8.1)	1.01 (−16.72, 18.73)	0.91
***Endline characteristics***			
Age, months	30.8 ± 1.8	0.53 (−2.12, 3.18)	0.69
Breastfeeding reported at 6 months (n = 293)			
Exclusive	77 (26.3)	ref	
Mixed	209 (71.3)	0.92 (−10.14, 11.98)	0.87
None	7 (2.4)	10.38 (−22.37, 43.13)	0.53
Breastfeeding reported until ≥12 months of age (n = 294)	257 (87.4)	−3.84 (−18.38, 10.70)	0.60
Dietary diversity, past day^1^			
< 4 food groups	117 (39.1)	ref	
≥ 4 food groups	182 (60.9)	−11.78 (−21.57, −2.00)	0.02*
Diarrhea, past 2 weeks	81 (27.1)	13.87 (3.15, 24.60)	0.01*
Fever, past 2 weeks	140 (46.8)	0.86 (−8.80, 10.51)	0.86
Difficulty breathing, past 2 weeks	201 (67.2)	5.44 (−4.80, 15.69)	0.30
Caregiver engagement, past 3 days^2^			
0-1	40 (13.4)	ref	
2-3	94 (31.4)	−10.38 (−26.07, 5.30)	0.19*
≥ 4	165 (55.2)	−12.23 (−26.87, 2.41)	0.10*
Number of toys/children’s books	6.4 ± 10.0	−0.56 (−1.04, −0.08)	0.02*

^1^ Based on consumption of the following 7 food groups: grains, roots, and tubers; pulses, nuts, and seeds; dairy products; flesh foods; eggs; vitamin-A-rich fruits and vegetables; and other fruits and vegetables.

^2^ Based on engagement in the following 6 activities: reading books, telling stories, singing songs, going outside the home, playing, and naming/counting/drawing.

*p < 0.20.

Abbreviations: CI, confidence interval; SD, standard deviation; SQ-LNS, small-quantity lipid-based nutrient supplements.

### Univariable analyses

The univariable associations between household, caregiver, and child predictors and SRT are also presented in Table 1. Household and caregiver characteristics measured at baseline and found to be significant predictors of SRT at the p < 0.05 level in univariable models included using charcoal/wood vs. electricity as a fuel source (β: 14.47, 95% CI: 1.55, 27.38, p = 0.03) and caregiver’s having senior secondary school or more vs. primary school or less (β: −13.72, 95% CI: −25.60, −1.83, p = 0.02) indicating that children in households using charcoal/wood had slower SRT (longer reaction times), while children whose caregivers had senior secondary school or more had faster SRT.

Significant child characteristics included LAZ at baseline (β: −3.64, 95% CI: −6.99, −0.30, p = 0.03), with higher LAZ associated with faster SRT; consuming ≥4 food groups vs. < 4 food groups in the past day (β: −11.78, 95% CI: −21.57, −2.00, p = 0.02), with more diverse diets associated with faster SRT; having diarrhea in the past 2 weeks (β: 13.87, 95% CI: 3.15, 24.60, p = 0.01), with recent diarrhea associated with slower SRT; and total number of toys/children’s books owned (β: −0.56, 95% CI: −1.04, −0.08, p = 0.02), with greater toy/book ownership associated with faster SRT.

In addition to these, being the only child <5 in the household, having piped water vs. other, assignment to the SQ-LNS vs. control study group, caregiver marital status, and caregiver engagement were found to be significant predictors at the p < 0.20 level and thus retained in the multivariable model. Notably, while WAZ at baseline was significant at the p < 0.20 level, it was not retained in the multivariable model due to its correlation with LAZ at baseline.

### Multivariable analysis

Results of the multivariable regression analysis are presented in Table 2. Characteristics found to be significant predictors of SRT at the p < 0.05 level in the multivariable model included being the only child <5 in the household (β: −10.02, 95% CI: −19.71, −0.33, p = 0.04), associated with faster SRT; LAZ at baseline (β: −3.17, 95% CI: −6.31, −0.04, p = 0.047), with higher LAZ associated with faster SRT; consuming ≥4 food groups in the past day (β: −10.42, 95% CI: −19.98, −0.86, p = 0.03), associated with faster SRT; and having diarrhea in the past 2 weeks (β: 12.38, 95% CI: 0.71, 24.06, p = 0.04), associated with slower SRT. No other predictors remained significant at the p < 0.05 level in the multivariable model. All VIF values were < 3, indicating no meaningful multicollinearity among predictors.

**Table 2:** Predictors of saccadic reaction time among young children in Lusaka, Zambia.

	*B (95% CI)*^*1*^ n = 294	*p-value*
**Household and caregiver baseline characteristics**		
Child is only household member <5 years	−10.02 (−19.71, −0.33)	0.04*
Water source		
Other	ref	
Piped	−11.19 (−31.52, 9.15)	0.28
Fuel source		
Electricity	ref	
Charcoal/wood	4.01 (9.63, 17.65)	0.56
Study group		
Control	ref	
Charts	9.58 (−5.13, 24.29)	0.20
SQ-LNS	11.77 (−2.99, 26.52)	0.12
Charts + SQLNS	3.65 (−10.73, 18.03)	0.62
Caregiver educational attainment		
Primary school or less	ref	
Junior secondary school	−3.43 (−17.70, 10.83)	0.64
Senior secondary school or more	−8.11 (−20.32, 4.10)	0.19
Caregiver marital status		
Married	ref	
Divorced, separated, or widowed	8.32 (−5.61, 22.25)	0.24
Never married	−8.58 (−20.95, 3.78)	0.17
**Child baseline characteristics**		
Child age	0.54 (−2.06, 3.13)	0.68
Child sex, female	2.53 (−7.25, 12.30)	0.51
Length-for-age Z-score	−3.17 (−6.31, −0.04)	0.047*
**Child endline characteristics**		
Dietary diversity, past day		
< 4 food groups	ref	
≥ 4 food groups	−10.42 (−19.98, −0.86)	0.03*
Diarrhea, past 2 weeks	12.38 (0.71, 24.06)	0.04*
Caregiver engagement, past 3 days		
0-1	ref	
2-3	−1.52 (−19.15, 16.11)	0.87
≥ 4	−3.98 (−21.06, 13.10)	0.90
Number of toys/children’s books	−0.28 (−0.69, 0.12)	0.17

^1^ Values obtained from a multivariable regression model of all predictors significant at the p < 0.20 level in addition to child age and sex.

*p < 0.05.

Abbreviations: CI: confidence interval; SQ-LNS, small-quantity lipid-based nutrient supplements.

### Correlations among measures of child development

Table 3 presents mean ± SD and Pearson correlation coefficients for endline measures of ECD, including SRT, GSED DAZ, HAZ, and HCZ. Mean ± SD was 313.38 ± 42.27 for SRT, 0.22 ± 1.43 for GSED DAZ, −1.98 ± 1.05 for HAZ, and 0.71 ± 1.06 for HCZ. SRT was significantly negatively correlated with HAZ (−0.176, p < 0.01) and HCZ (−0.132, p < 0.05), indicating that children with higher HAZ and HCZ had faster SRT. No significant correlation was observed between SRT and GSED DAZ. GSED DAZ was significantly positively correlated with HAZ (0.153, p < 0.01) and HCZ (0.182, p < 0.01). Finally, the strongest correlation was observed between HAZ and HCZ (0.384, p < 0.001).

**Table 3:** Correlation coefficient matrix of endline child development indicators among young children in Lusaka, Zambia^1^.

	*Mean ± SD*	SRT	GSED DAZ	HAZ	HCZ
SRT	313.38 ± 42.27	1.00			
GSED DAZ	0.22 ± 1.43	−0.038	1.00		
HAZ	−1.98 ± 1.05	−0.176**	0.153**	1.00	
HCZ	0.71 ± 1.06	−0.132*	0.182**	0.384***	1.00

^1^ Values are Pearson correlation coefficients.

* p < 0.05, **p < 0.01, ***p < 0.001.

Abbreviations: DAZ, development-for-age Z-score; GSED, Global Scales for Early Development; HAZ, height-for-age Z-score; HCZ, head circumference-for-age Z-score; SD, standard deviation; SRT, saccadic reaction time.

## Discussion

In this secondary analysis of a randomized controlled trial, we examined a number of household, caregiver, and child characteristics as predictors of SRT as well as the correlation between SRT and other measures of ECD among young children ~31 months of age in Lusaka, Zambia. Overall, using multivariable regression modeling, we identified several significant predictors of SRT in this population, including birth spacing, LAZ at baseline, dietary diversity, and diarrheal disease. Furthermore, we found SRT to be significantly negatively correlated with HAZ and HCZ; however, correlations were relatively weak at −0.176 and −0.132, respectively.

SRT is a measure of visual processing speed with faster times indicative of improved developmental outcomes. In a study by Dougherty & Haith, faster saccadic latencies at 3.5 months were associated with higher intelligence at 4 years (including verbal, performance, and full-scale IQ) [15]. Another study by Rose et al. found an association between visual reaction times at 7 and 12 months and executive functioning (i.e., working memory and flexible switching of attention) at 11 years [16]. Mean SRT in our study (~313 ms) was similar to findings from high-income settings, where averages have ranged from 270 to 375 ms [27–30]. Among infants in Malawi, mean SRT was found to be ~ 389 ms at ~9 months of age [17], with slower times potentially due to the younger age at assessment [31]. However, it is important to note that comparing absolute SRTs across studies is challenging due to methodological differences, including variations in setup, stimulus materials, and participant age.

In terms of predictors of SRT, our findings contrast with those of Pyykkö et al., who reported no significant associations between visual attention measures and gestational age, nutritional status, or aspects of the rearing environment (such as maternal cognition, psychosocial well-being, socioeconomic status, and care practices) among infants in rural Malawi [32]. While a study by Leppänen et al. found SRT to be negatively correlated with household wealth (assessed by the ownership of various assets) in South Africa and Zambia, no other predictors were examined [19].

Numerous studies have linked short interpregnancy intervals to an increased risk of stunting in children <5 years of age in LMICs [33–36]. In this study, we found longer intervals (i.e., being the only child <5 years in the household at baseline) to be associated with faster SRT at endline, which adds to the limited research on birth intervals and more direct measures of ECD in LMICs. In a pooled analysis of Demographic and Health Survey (DHS) data from 13 countries, longer birth intervals (≥33 months) were associated with improved socio-emotional development [relative risk (RR): 1.04, 95% CI: 1.00, 1.09], but not cognitive development (RR: 1.02, 95% CI: 0.98, 1.06), assessed via the Early Childhood Development Index. Although the authors were unable to identify the mechanisms behind their findings, they suggest longer birth intervals likely influence both biological and behavioral mechanisms, including increased parental investment and reduced sibling competition for parental resources [37].

Similarly, while dietary diversity has a well-established link to stunting [38–40], there are relatively fewer studies examining dietary diversity and its association with more direct measures of ECD. However, similar to our findings, a limited number of observational studies have demonstrated that diet quality, including increased dietary diversity, is positively associated with ECD, likely due to increased exposure to nutrients critical for brain development [41–44]. Finally, we found diarrhea within the past 2 weeks, but not other morbidities, to be a significant predictor of SRT. While the exact mechanisms have not been elucidated, diarrhea can cause chronic inflammation and reduce the ability of the gut to absorb nutrients critical for brain development [45]. Notably, previous studies have shown that diarrhea alone, independent of malnutrition, can contribute to poor ECD [46].

Our findings point to a significant, yet relatively weak correlation between SRT and commonly used anthropometric measurements of ECD, namely HAZ and HCZ. Notably, HAZ and HCZ also had a significant, yet relatively weak correlation with GSED DAZ. Together, these results support previous studies which have shown that child growth is not a sensitive and therefore suitable indicator of ECD [9]. Furthermore, we found no significant correlation between SRT and GSED DAZ, which was designed to reflect children’s overall development across multiple domains, including cognitive, motor, language, and social-emotional [21,47]. This suggests that SRT, which primarily aims to assess cognitive function, may not be a holistic measure of ECD. Unfortunately, the GSED does not produce domain-specific development scores that would allow us to examine the correlation between SRT and cognitive development in this sample.

Overall, these findings raise important questions regarding how eye-tracking assessments should be conceptualized and utilized in the context of existing assessments of child development. The relatively weak correlations observed between SRT and anthropometric measurements, as well as the lack of correlation with GSED DAZ, suggest that SRT is not simply a direct proxy for commonly used measures of physical growth or developmental status. Rather, SRT may capture a more specific aspect of neurocognitive functioning by offering a direct, performance-based assessment of real-time cognitive processing that is less dependent on language, caregiver perception, or cultural context. As such, SRT may be best viewed as a complementary measure that can enrich existing assessment approaches rather than replace them.

Our study has a number of strengths. To our knowledge, this is one of only a few studies where eye-tracking assessments were conducted in an LMIC setting and one of the first to examine significant predictors of SRT [17–19,32]. Furthermore, children participating in this study were enrolled in a rigorous cluster-randomized trial with detailed household questionnaire conducted at baseline and endline, allowing us to examine a number of household, caregiver, and child predictors. SRT measurements are relatively precise and unbiased, and the assessments proved relatively non-invasive and well accepted in this setting. Finally, in addition to SRT, additional measures of ECD were collected in this study, including HAZ, HCZ, and GSED DAZ, allowing us to assess correlations among different ECD assessment methods.

With regard to limitations, the study included only children 27–35 months from Lusaka district, an area that covers the urban population of the capital city, limiting the generalizability of our findings to children in other age groups or those living in rural or peri-urban areas of Zambia, where exposures may differ. Additionally, only ~50% of caregiver-child pairs from Lusaka district chose to participate in the biomarkers sub-study. Although no statistically significant differences in background characteristics were observed between those who participated and those who did not, there remains the possibility of selection bias related to unmeasured factors. Furthermore, data for this study were derived from baseline and endline questionnaires, with no longitudinal follow-up between these time points, limiting our ability to assess relationships over time. Finally, given the exploratory nature of the study, no adjustments were made for multiple comparisons, which increases the risk of type I error and may overestimate the significance of some findings.

In conclusion, our results contribute to the growing body of literature supporting the use of eye-tracking assessments in LMIC settings and suggest that SRT may capture a distinct dimension of early cognitive function that is not fully reflected by traditional growth or development measures. By identifying key modifiable biological and environmental risk factors to poor ECD, such as inadequate birth spacing, poor nutrition, and infectious disease, this study advances understanding of the factors shaping early cognitive development and highlights the potential role of eye-tracking as a complementary tool for ECD research and program evaluation in LMIC settings.

## Supporting information

S1 File Data. (XLSX)
